# Mathematical modeling for bioprocess optimization of a protein drug, uricase, production by *Aspergillus welwitschiae* strain 1–4

**DOI:** 10.1038/s41598-019-49201-1

**Published:** 2019-09-10

**Authors:** Noura El-Ahmady El-Naggar, S. A. Haroun, Eman M. El-Weshy, E. A. Metwally, A. A. Sherief

**Affiliations:** 1 Department of Bioprocess Development, Genetic Engineering and Biotechnology Research Institute, City of Scientific Research and Technological Applications, Alexandria, 21934 Egypt; 2 Department of Botany, Faculty of Science, Mansoura University, Mansoura, Egypt

**Keywords:** Oxidoreductases, Applied microbiology

## Abstract

Microbial uricase is effective protein drug used to treat hyperuricemia and its complications, including chronic gout, also in prophylaxis and treatment of tumor lysis and organ transplants hyperuricemia. Uricase is commonly used as diagnostic reagent in clinical analysis for quantification of uric acid in blood and other biological fluids. Also, it can be used as an additive in formulations of hair coloring agents. A newly isolated strain, *Aspergillus* sp. 1–4, was able to produce extracellular uricase on a medium containing uric acid as inducer. Phylogenetic analysis based on ITS region sequence analysis and phenotypic characteristics showed that *Aspergillus* sp. strain 1–4 is closely related to *Aspergillus welwitschiae* and its nucleotide sequence was deposited in the GenBank database and assigned sequence accession number MG323529. Statistical screening using Plackett-Burman design with 20 runs was applied to screen fifteen factors for their significance on uricase production by *Aspergillus welwitschiae*. Results of statistical analysis indicated that incubation time has the most significant positive effect on uricase production followed by yeast extract and inoculum size with the highest effect values of 13.48, 5.26 and 4.75; respectively. The interaction effects and optimal levels of these factors were evaluated using central composite design. The maximum uricase production was achieved at incubation time (5 days), yeast extract (2 g/L) and inoculum size (4 mL/50 mL medium) are the optimum levels for maximum uricase production (60.03 U/mL). After optimization, uricase production increased by 3.02-folds as compared with that obtained from the unoptimized medium (19.87 U/mL).

## Introduction

In all cells of the human body, uric acid is the end product of purine nucleotide metabolism, and is excreted out of the body via the kidneys in the urine. Uric acid normally found in the blood and tissues are derived from both degradation of purine containing foods in normal diets especially red meats and organ meats (such as liver and kidneys), fructose-sweetened drinks, as well as seafood and consumption of alcohol^1^ and from the breakdown of old cells. Overproduction and accumulation of uric acid crystals in humans blood stream over the normal value (hyperuricemia, due to the absence of human uricase) can promote a painful metabolic disorder disease known as gout^2^, form kidney stones and subsequent urate nephropathy^3^ and renal failure^4^, idiopathic calcium urate nephrolithiasis^5^. It was also reported that uric acid levels are often elevated in pediatric patients with acute lymphoblastic leukemia^6^ and toxemia of pregnancy^7^.

Uricase (urate oxidase, urate: oxygen oxido-reductase, EC 1.7.3.3) is the enzyme which catalyzes the uric acid oxidation to more water-soluble, poorly toxic and rapidly excreted by the kidneys, allantoin, hydrogen peroxide and carbon dioxide^8^ and plays an important role in nitrogen metabolism.

$${\rm{Uric}}\,{\rm{acid}}+{{\rm{H}}}_{2}{\rm{O}}+{{\rm{O}}}_{{\rm{2}}}\,\mathop{\longrightarrow }\limits^{{\rm{Uricase}}}\,{\rm{Allantoin}}+{{\rm{H}}}_{2}{{\rm{O}}}_{2}+{{\rm{CO}}}_{2}$$

The first important uricase application was in the clinical analysis as a diagnostic reagent for uric acid determination in blood and other biological fluids by coupling it with 4-amino-antipyrine-peroxidase system^9,10^. Uricase is used as a protein drug to reduce the accumulation of toxic urate, in the treatment of hyperuricemia and gout, as well as in prophylaxis and treatment of tumor lysis hyperuricemia^11,12^. Immobilized uricase can be used as a uric acid biosensor. Administration of uricase was found to be more potent; it reduced the elevated serum urate concentrations more efficiently compared to other urate-lowering therapies like allopurinol^13^. Rasburicase has been effectively used for the prevention and treatment of hyperuricemia caused by tumor lysis and organ transplants^14^. It is also used as an additive in commercial formulations of hair coloring agents^15^.

Microorganisms, higher plants and animals are able to produce uricase on their own, but uricase cannot be produced by humans. Microorganisms have proven to be a very efficient and economical source of uricase because of their economic cultivation, optimization and purification, thus facilitating the microbial production of uricase. Some microorganisms such as *Gliocladium viride*^16^, *Pseudomonas putida*^17^ and *Nocardi farcinica*^18^ have been used to produce uricase. Despite uricase being obtained from several sources, its growing importance in the therapy and diagnosis necessitates the search for new microbial producers to produce uricase with better enzymatic properties and better yield^19^.

Uricase production by many microorganisms are strongly controlled by medium components and cultural parameters and by their ability to degrade and use uric acid for growth. Optimization of the medium components and culture condition parameters is the main objective of the biological processes where it has a powerful impact on the production of microbial uricase, as it may effect on product concentration, and the cost of downstream product separation^20^.

The conventional method of optimization is one factor-at-a-time optimization technique, where one factor is optimized by changing it while maintaining the other factors at constant level. The conventional optimization technique has various disadvantages such as high cost, difficulty, time consuming and neglecting the interactions among different variables^21^. For several decades, well-defined approaches of statistical experimental designs have been effectively used to optimize different nutritional components and environmental conditions to achieve optimal production and to predict response values in relation to the experimental factors^22^. Response surface methodology (RSM) is an effective technique for optimizing the system of multiple variables, for predicting the optimal conditions with a few experiments, for clarifying the interactive effects of the tested variables on the response, for minimizing the time and costs involved in the study and for avoiding the misinterpretation occurring in one factor at a time optimization^23,24^.

The objectives of the present work were to determine the potentiality of the newly isolated *Aspergillus* sp. strain 1–4 for uricase production, to identify the fungal strain and to optimize uricase production by *Aspergillus welwitschiae* using Plackett–Burman and central composite designs.

## Results and Discussion

### Potentiality of *Aspergillus* sp. strain 1–4 for uricase production

The extracellular uricase producing microorganisms convert the suspended insoluble, white crystals of uric acid in the medium used for agar plate assay method to water soluble allantoin, thus producing a clear zone around the colonies. The produced clear zone diameter is directly linked to the extracellular uricase production, meaning a larger clear zone diameter indicated a greater activity of extracellular uricase^25^. The formation of clear zone around the fungal colony of *Aspergillus* sp. strain 1–4 indicated its ability to produce uricase. Uricase activity obtained by *Aspergillus* sp. strain 1–4 under submerged fermentation condition was found to be 19.87 U/mL.

### Scanning electron microscopy for *Aspergillus* sp. strain 1–4

**Figure 1:**
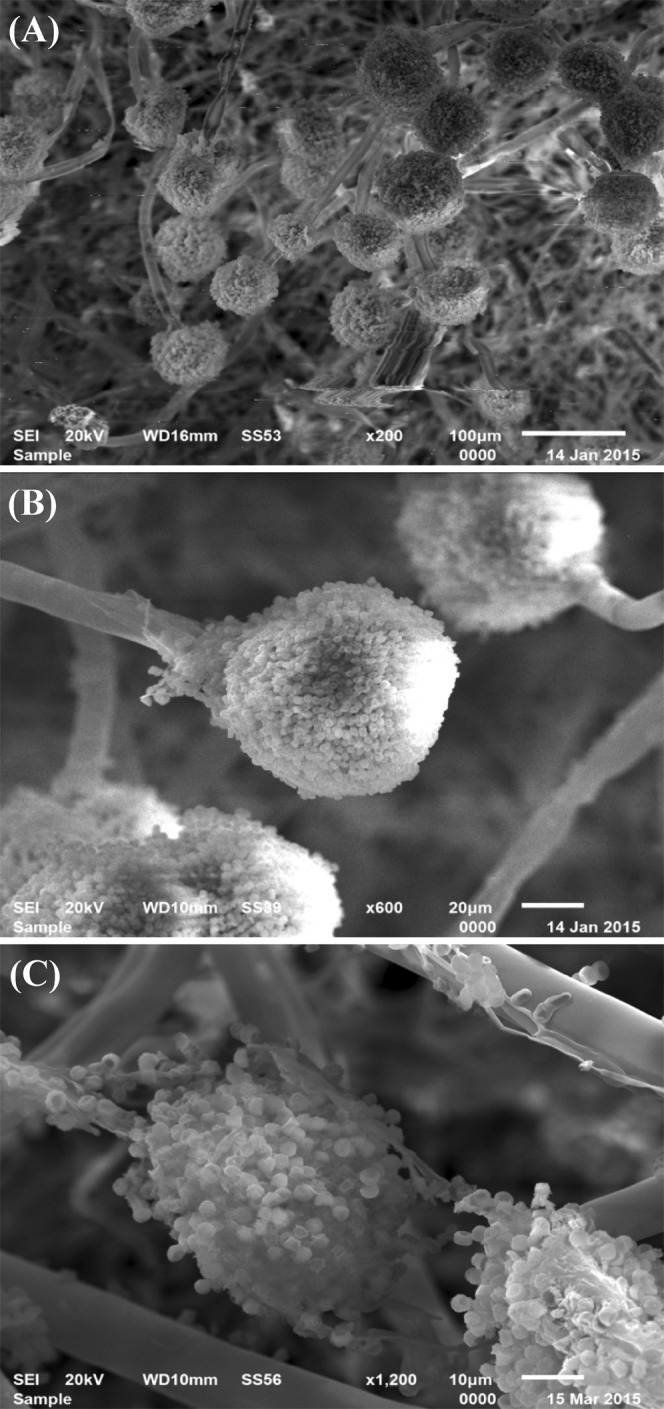
Scanning electron micrographs of *Aspergillus* sp. strain 1-4; (**A**–**C**) at different magnifications 200x, 600x and 1200x; respectively.

To determine structure and surface fine features of *Aspergillus* sp. strain 1–4, scanning electron microscope with high resolution was used at different magnifications 200x, 1600x and 1200x). Figure 1 shows the upright conidiophores bearing conidial heads and chains of spherical, rough walled spherical conidia.

### Molecular identification for *Aspergillus* sp. strain 1–4

**Figure 2:**
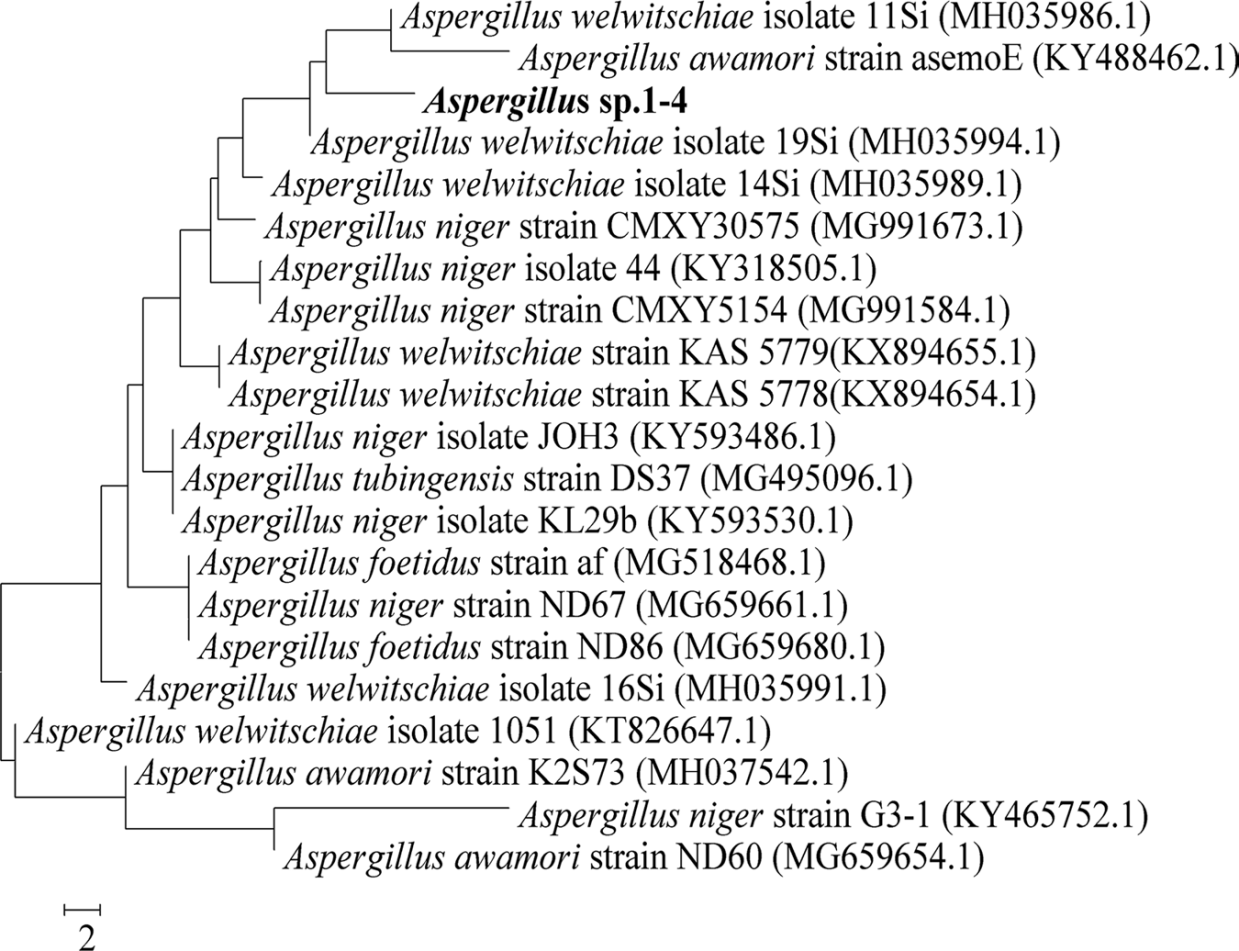
Phylogenetic tree obtained by neighbor-joining analysis of 18S ribosomal RNA gene (partial), internal transcribed spacer 1, 5.8S ribosomal RNA gene, internal transcribed spacer 2 and 28S ribosomal RNA gene (partial), showing the position of *Aspergillus* sp. within the genus *Aspergillus*. GenBank sequence accession numbers are indicated in parentheses after the strain names.

The PCR product of the amplified 18S rRNA fragment with ITS1 and ITS4 primers resulted in approximately 535 bp fragment (Supplementary Fig. S1). The obtained sequence of *Aspergillus* sp. strain 1–4 was compared with those nucleotide sequences collected from the GeneBank database by using BLAST^26^. The sequenced product was deposited under accession number MG323529 in the GenBank database. The phylogenetic tree (Fig. 2) was built using neighbor-joining method of Saitou and Nei^27^, demonstrating the position of *Aspergillus* sp. strain 1–4 within the genus *Aspergillus*. Based on the phylogenetic analysis along with the phenotypic characteristics, the *Aspergillus* sp. strain 1–4 has been identified as *Aspergillus welwitschiae* strain 1–4.

### Screening of the significant variables for uricase production by *Aspergillus welwitschiae* using Plackett-Burman design

The statistical experimental design of Plackett-Burman (fractional factorial design) is used to identify the most significant independent variables when the researcher encounters a large number of variables and he is not certain that the variables are best for producing maximum response^28^. The effects of different fifteen factors were studied using the statistical experimental design of Plackett-Burman to identify the most significant variables for optimization process to attain high uricase production. These factors consist of physical factors like (temperature, pH, inoculums size, inoculums age, incubation time and medium volume) and chemicals factors like (sucrose, uric acid, peptone, yeast extract, NaNO_3_, K_2_HPO_4_, NaCl, MgSO_4_.7H_2_O and FeSO_4_.7H_2_O) as in Table 1. The low (−1) and high (+1) levels selected for the investigated fifteen factors are given in Table 1. The design matrix with the different levels of variables and a set of twenty experiments to determine the production of uricase under different combinations of variables and the corresponding uricase production are given in Table 1. The data in Table 1 show a great variation in the uricase production in the 20 trials of Plackett-Burman design, which range from 20.92 to 58.21 U/mL. This variation is due to the presence of different combinations with the different levels of factors.

**Table 1:** Plackett–Burman design at two levels applied to select the factors that significantly affect uricase production by *Aspergillus welwitschiae*.

Run no.	Coded levels of the selected independent variables																			Uricase activity (U/mL)		Residuals
	Incubation time	Temperature	pH	Inoculum size	Inoculums age	Medium volume	Sucrose	Peptone	Uric acid	Yeast extract	NaNO_3_	K_2_HPO_4_	MgSO_4_.7H_2_O	NaCl	FeSO_4_. 7H_2_O	D 1	D 2	D 3	D4	Actual	Predicted	
1	1	1	−1	−1	1	1	−1	1	1	−1	−1	−1	−1	1	−1	1	−1	1	1	48.2	49.22	−1.02
2	−1	1	1	−1	−1	−1	−1	1	−1	1	−1	1	1	1	1	−1	−1	1	1	20.92	22.17	−1.25
3	−1	−1	−1	−1	1	−1	1	−1	1	1	1	1	−1	−1	1	1	−1	1	1	46.38	47.40	−1.02
4	1	1	1	1	−1	−1	1	1	−1	1	1	−1	−1	−1	−1	1	−1	1	−1	58.21	58.95	−0.74
5	−1	−1	1	−1	1	−1	1	1	1	1	−1	−1	1	1	−1	1	1	−1	−1	31.83	30.58	1.25
6	−1	1	−1	1	−1	1	1	1	1	−1	−1	1	1	−1	1	1	−1	−1	−1	30.01	30.88	−0.87
7	−1	1	1	−1	1	1	−1	−1	−1	−1	1	−1	1	−1	1	1	1	1	−1	30.92	29.54	1.38
8	1	1	1	−1	−1	1	1	−1	1	1	−1	−1	−1	−1	1	−1	1	−1	1	58.21	57.47	0.74
9	1	1	−1	1	1	−1	−1	−1	−1	1	−1	1	−1	1	1	1	1	−1	−1	57.29	56.04	1.25
10	1	−1	1	−1	1	1	1	1	−1	−1	1	1	−1	1	1	−1	−1	−1	−1	47.29	48.40	−1.11
11	−1	1	1	1	1	−1	−1	1	1	−1	1	1	−1	−1	−1	−1	1	−1	1	42.74	42.00	0.74
12	1	−1	1	1	1	1	−1	−1	1	1	−1	1	1	−1	−1	−1	−1	1	−1	47.29	48.27	−0.98
13	−1	1	−1	1	1	1	1	−1	−1	1	1	−1	1	1	−1	−1	−1	−1	1	40.01	41.39	−1.38
14	1	−1	−1	1	1	−1	1	1	−1	−1	−1	−1	1	−1	1	−1	1	1	1	43.83	42.96	0.87
15	1	1	−1	−1	−1	−1	1	−1	1	−1	1	1	1	1	−1	−1	1	1	−1	44.20	43.05	1.15
16	1	−1	1	1	−1	−1	−1	−1	1	−1	1	−1	1	1	1	1	−1	−1	1	43.65	44.80	−1.15
17	−1	−1	−1	1	−1	1	−1	1	1	1	1	−1	−1	1	1	−1	1	1	−1	46.93	45.78	1.15
18	−1	−1	−1	−1	−1	−1	−1	−1	−1	−1	−1	−1	−1	−1	−1	−1	−1	−1	−1	29.1	30.21	−1.11
19	1	−1	−1	−1	−1	1	−1	1	−1	1	1	1	1	−1	−1	1	1	−1	1	40.92	39.94	0.98
20	−1	−1	1	1	−1	1	1	−1	−1	−1	−1	1	−1	1	−1	1	1	1	1	35.47	34.36	1.11
**Level**	**Days**	**°C**	**pH**	** mL/50 mL medium**	**Hour**	**mL**	**g/L**	**g/L**	**g/L**	**g/L**	**g/L**	**g/L**	**g/L**	**g/L**	**g/L**							
−1	4	25	6	1	48	25	20	0.5	3	0.5	0.5	1	0.2	0.2	0.01							
1	7	35	8	4	72	50	30	2	5	2	2	2	0.8	0.8	0.03							

“D, dummy”.

**Figure 3:**
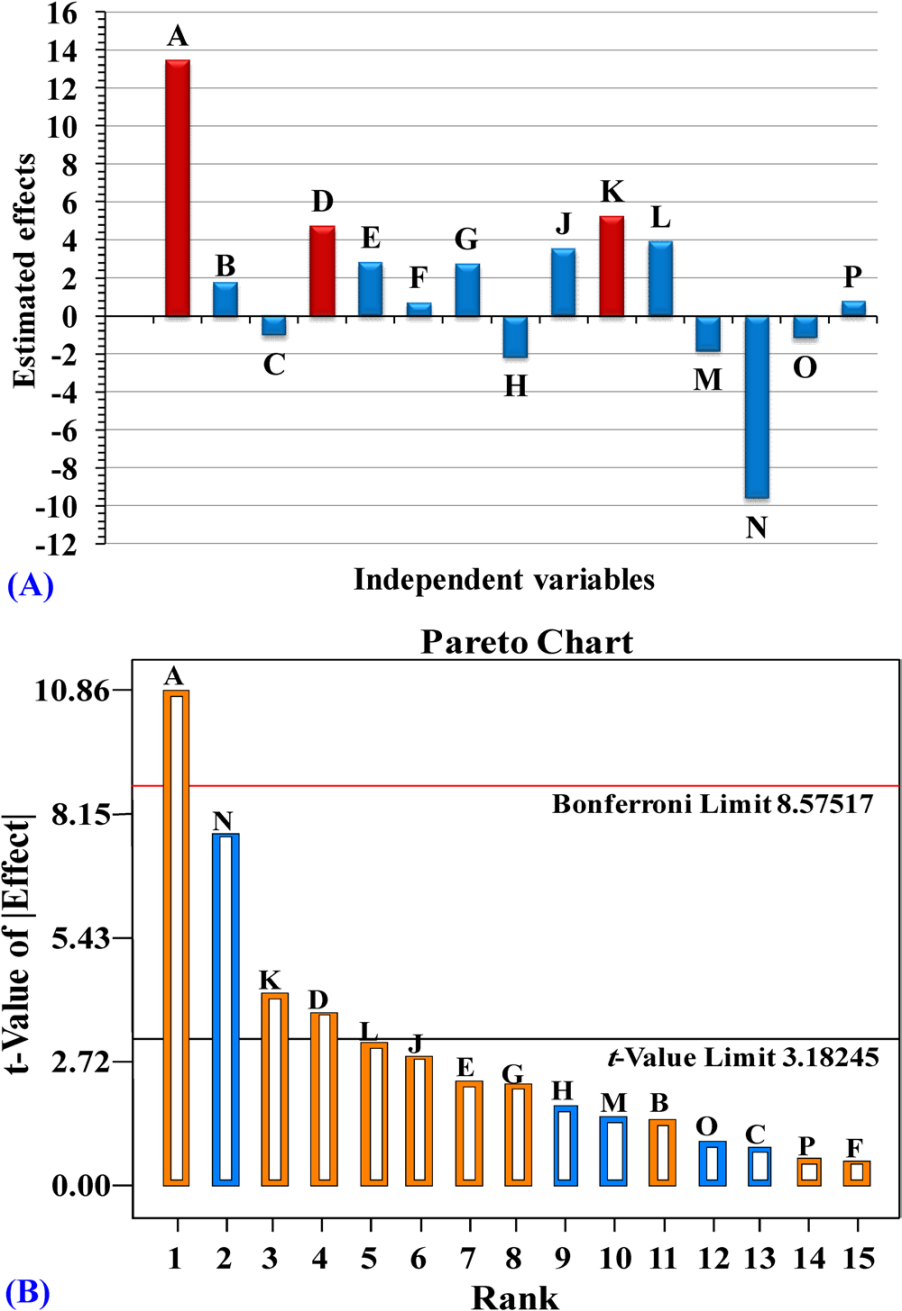
(**A**) Effect of independent variables on uricase production (The red color represent the independent variables with most significant positive effect on uricase production). (**B**) Pareto chart illustrates the order and significance of the variables affecting uricase production by *Aspergillus welwitschiae* using Plackett-Burman design (the blue color represent negative effects and the orange color represent positive effects).

In order to determine the relationship between the independent variables and uricase production by *Aspergillus welwitschiae*, the multiple-regression statistical analysis was performed and the results are presented in Tables 2 and 3. Table 2 and Fig. 3A shows the effect of independent variables on uricase production by *Aspergillus welwitschiae* using Plackett-Burman design. The large positive or negative effect indicates that the factor has a large impact on uricase production. While the near zero effect means that the factor has little or no effect on uricase production. Among the studied factors: temperature, incubation time, inoculums age, inoculums size, medium volume, uric acid, sucrose, yeast extract, NaNO_3_, and FeSO_4_. 7H_2_O concentrations were found to have positive effects on uricase production. On the other hand, pH, peptone, K_2_HPO_4_, MgSO_4_.7H_2_O and NaCl concentrations were found to have negative effects on uricase production.

**Table 2:** Coefficients, effects and the contributions percentages of the factors affecting production of uricase by *Aspergillus welwitschiae* using Plackett-Burman design.

Term	% Contribution	Coefficient	Effect
Intercept		42.17	
A-Incubation time (days)	46.90	6.74	13.48
B-Temperature (°C)	0.84	0.90	1.80
C-pH	0.28	−0.52	−1.03
D-Inoculum size (mL/50 mL medium)	5.82	2.37	4.75
E-Inoculums age (h)	2.05	1.41	2.82
F-Medium volume (mL/250 mL conical flask)	0.13	0.36	0.71
G-Sucrose (g/L)	1.95	1.37	2.75
H-Peptone (g/L)	1.21	−1.08	−2.16
J-Uric acid (g/L)	3.25	1.77	3.55
K-Yeast extract (g/L)	7.14	2.63	5.26
L-NaNO_3_ (g/L)	3.95	1.96	3.91
M-K_2_HPO_4_ (g/L)	0.87	−0.92	−1.84
N-MgSO_4_.7H_2_O (g/L)	23.91	−4.81	−9.62
O-NaCl (g/L)	0.36	−0.59	−1.18
P-FeSO_4_. 7H_2_O (g/L)	0.14	0.37	0.75

**Table 3:** Regression statistics and ANOVA (analysis of variance) for the Plackett-Burman design experimental results of uricase production by *Aspergillus welwitschiae*.

Source	Sum of squares	*Df*	Mean square	*F-*value	*P-*value
Model	1913.177	15	127.545	21.835	0.0044*
A	908.309	1	908.309	155.496	0.0002*
B	16.232	1	16.232	2.779	0.1708
C	5.340	1	5.340	0.914	0.3932
D	112.632	1	112.632	19.282	0.0118*
E	39.677	1	39.677	6.793	0.0597
F	2.519	1	2.519	0.431	0.5472
G	37.763	1	37.763	6.465	0.0638
H	23.410	1	23.410	4.008	0.1159
J	62.906	1	62.906	10.769	0.0305*
K	138.222	1	138.222	23.663	0.0083*
L	76.401	1	76.401	13.079	0.0224*
M	16.895	1	16.895	2.892	0.1642
N	463.088	1	463.088	79.277	0.0009*
O	6.997	1	6.997	1.198	0.3352
P	2.784	1	2.784	0.477	0.5279
Residual	23.365	4	5.841		
**Std. Dev**.	2.416	**R-Squared**		0.9879	
**Mean**	42.169	**Adj R-Squared**		0.9427	
**C.V.%**	5.731	**Adeq Precision**		17.0143	
**PRESS**	584.135				

*“Significant values, *df*: Degree of freedom, *P*: Level of significance, *F*: Fishers’s function, C.V: Coefficient of variation”.

Pareto chart (Fig. 3B) illustrate in descending order the variables influencing the production of uricase by *Aspergillus welwitschiae* using Plackett-Burman design that placed above and below the horizontal line (*t*-value limit). A high *t*-test value and a low probability indicated a high significance^29^. The standard Pareto chart consists of bars of length that equivalent to the absolute value of the estimated effects divided by the standard error. The bars are shown in accordance with effect value (*t*-value), with the highest effects at the first. The blue color represents the factors with negative effects (MgSO_4_.7H_2_O (N), peptone (H), K_2_HPO_4_ (M), NaCl (O) and pH (C)) with values (−9.62, −2.16, −1.84, −1.18 and −1.03; respectively) and the orange color represents the factors with positive effects (incubation time (A), yeast extract (K), inoculum size (D), NaNO_3_ (L), uric acid (J), inoculums age (E), sucrose concentration (G), temperature (B), FeSO_4_. 7H_2_O (P) and medium volume (F)) with coefficient values (13.48, 5.26, 4.75, 3.91, 3.55, 2.82, 2.75, 1.80, 0.75 and 0.71; respectively).

Table 3 shows regression analysis and ANOVA (the analysis of variance) for the Plackett-Burman design experimental results of uricase production by *Aspergillus welwitschiae*. The model *F*-value is 21.835, which implies that the model is significant. On the other hand; the *P-*value used as a tool to verify the significance of each variable; the small *P-*value indicates a significant effect of the independent variable^30^. *P*-values < 0.05 means that the model terms are significant. Variables at confidence levels greater than 95% (*P* < 0.05) were considered significant^31^. Some investigators reported that confidence levels higher than 70% are acceptable^32^. In this case, incubation time (A), MgSO_4_.7H_2_O (N), yeast extract (K) and inoculum size (D), NaNO_3_ (L) and uric acid (J) were most significant factors affecting uricase production with the lower probability values (0.0002, 0.0009, 0.0083, 0.0118, 0.0224 and 0.0305; respectively). Among the most significant factors: incubation time, yeast extract, inoculums size, NaNO_3_ and uric acid concentrations were found to have positive effects on uricase production. While, MgSO_4_.7H_2_O concentration was found to has negative effect on uricase production.

The suitability of the model and the variability of the response explained by the independent variables can be verified with the value of R^2^ “determination coefficient”. A regression model that has an R^2^-value larger than 0.9 is considered to have a very high correlation^33^. The regression model in our study has R^2^ = 0.9879 which means that 98.79% of variation in uricase production can be explained by the independent variables and only 1.21% of the total changes are not explained by the independent variables. This means that the regression model provides an excellent explanation for the relationship between uricase production and the independent variables. In addition, the Adjusted R^2^ of 0.9427 is very high to prove the accuracy of the model and is good to calculate uricase production. Also, the adjusted R^2^ implies a high correlation between the predicted and the observed values. The value of C.V. (5.731%) indicates a large precision of the experiment. The value of PRESS is 584.135 and the values of standard deviation and mean are 2.416 and 42.169; respectively (Table 3).

Regression equation in terms of coded factors:

1 $$\begin{array}{ccc}{\rm{R}}{\rm{e}}{\rm{s}}{\rm{p}}{\rm{o}}{\rm{n}}{\rm{s}}{\rm{e}}\,({\rm{u}}{\rm{r}}{\rm{i}}{\rm{c}}{\rm{a}}{\rm{s}}{\rm{e}}\,{\rm{a}}{\rm{c}}{\rm{t}}{\rm{i}}{\rm{v}}{\rm{i}}{\rm{t}}{\rm{y}},\,{\rm{U}}/{\rm{m}}{\rm{L}}) & = & 42.17+6.74{\rm{A}}+0.9{\rm{B}}\\  &  & -\,0.52{\rm{C}}+2.37{\rm{D}}+1.41{\rm{E}}+0.36{\rm{F}}\\  &  & +\,1.37{\rm{G}}-1.08{\rm{H}}+1.77{\rm{J}}+2.63{\rm{K}}\\  &  & +\,1.96{\rm{L}}-0.92{\rm{M}}-4.81{\rm{N}}-0.59{\rm{O}}+0.37{\rm{P}}\end{array}$$

where A, B, C, D, E, F, G, H, J, K, L, M, N, O and P are “incubation time, temperature, pH, inoculum size, inoculum age, medium volume, sucrose, peptone, uric acid, yeast extract, NaNO_3_, K_2_HPO_4_, MgSO_4_.7H_2_O, NaCl and FeSO_4_.7H_2_O concentrations”; respectively.

### Model adequacy checking

**Figure 4:**
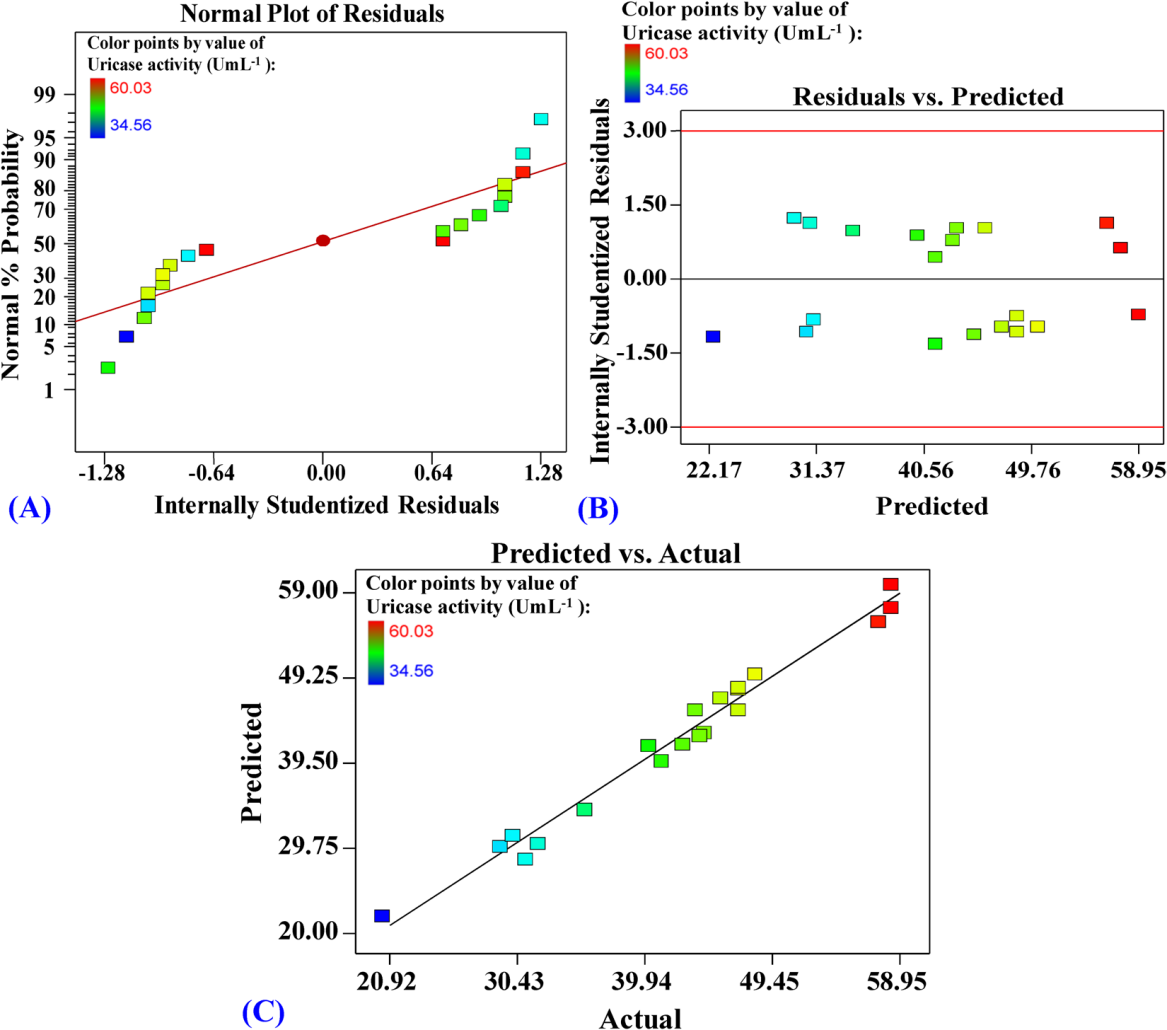
(**A**) The normal probability plot of the studentized residuals. (**B**) Plot of residuals against predicted values. (**C**) Plot of predicted vs. actual values for uricase production by *Aspergillus welwitschiae* determined by the first-order polynomial equation.

The normal probability plot (NPP) is a tool to indicate if the residuals follow a normal distribution; the data are plotted against a theoretical normal distribution. The normal probability plot of the studentized residuals (Fig. 4A), shows that the plotted points are close to a straight line that indicates the model has been well fitted with the experimental results. A plot of residuals versus the predicted response (Fig. 4B) is a scatter plot of residuals on the y-axis against predicted response values on the x-axis. Figure 4B shows the residuals are randomly scattered around the horizontal zero reference indicating a good fit of the model for uricase production by *Aspergillus welwitschiae*. The relationship between predicted and actual (experimental) values of uricase production by *Aspergillus welwitschiae* is presented in Fig. 4C that indicates the model is sufficient to illustrate uricase activity by *Aspergillus welwitschiae*.

In a confirmation experiment, the medium of the following composition g/L: sucrose 30, peptone 0.5, uric acid 5, NaNO_3_ 0.5, yeast extract 2, MgSO_4_.7H_2_O 0.2, K_2_HPO_4_ 1, NaCl 0.2 and FeSO_4_.7H_2_O 0.03, medium volume 50 mL/250 mL flask, inoculum size 1% (v/v), incubation time 7 days, inoculum age 48 h, incubation temperature 35 °C and pH 8 was used. Under the previously mentioned medium composition and physical conditions, the obtained experimental uricase activity was 58.21 U/mL which is greater than uricase activity obtained using the basal medium prior to applying Plackett-Burman design by 2.92 times (19.87 U/mL). The experimental uricase activity (58.21 U/mL) was close to the predicted value of 57.47 U/mL. This revealed a high degree of precision (98.72%); its percentage error is 1.28%.

### Optimization of significant variables affecting uricase production by *Aspergillus welwitschiae* using central composite design (CCD)

The most significant variables positively affect uricase production as identified by Plackett–Burman design results (incubation time, inoculum size and yeast extract with contribution percentages of 46.90, 5.82, and 7.14%; respectively) were further subjected to the central composite design to identify possible interactions between these factors and to determine optimal levels of these variables to produce maximum uricase production by *Aspergillus welwitschiae*.

Table 4 shows central composite design matrix that present the uricase production by *Aspergillus welwitschiae* in the 20 experiments of the design as influenced by incubation time (days) (X_1_), inoculum size (mL/50 mL medium) (X_2_) and yeast extract (g/L) (X_3_) in addition to the predicted uricase production and the residuals. Each of the significant variables was taken at a central coded value of zero and evaluated at five coded levels “−1.68, −1, 0, +1, +1.68”. The response value (Y) in each experiment was uricase production by *Aspergillus welwitschiae*. *Aspergillus welwitschiae* uricase activties obtained by the experiments were ranged from 34.56 to 60.03 U/mL. The highest level of uricase production was obtained in run no. 2 with value 60.03 U/mL where the incubation time was 5 days, inoculum size was 0.08 (v/v) and yeast extract concentration was 2 g/L.

**Table 4:** Central composite design representing uricase production by *Aspergillus welwitschiae* as affected by incubation time (days) (X_1_), inoculum size (mL/50 mL medium) (X_2_) and yeast extract (g/L) (X_3_) with actual and coded factor levels.

Std	Run	Type	Variables			Uricase activity (U/mL)					Residuals
			X_1_	X_2_	X_3_	Experimental			Predicted		
8	1	Factorial	1	1	1	36.28			39.61		−3.33
15	2	Center	0	0	0	60.03			58.08		1.95
5	3	Factorial	−1	−1	1	38.19			38.41		−0.22
14	4	Axial	0	0	1.68	34.56			32.82		1.74
17	5	Center	0	0	0	55.47			58.08		−2.61
12	6	Axial	0	1.68	0	42.33			40.29		2.04
13	7	Axial	0	0	−1.68	40.02			40.82		−0.80
18	8	Center	0	0	0	57.29			58.08		−0.79
20	9	Center	0	0	0	58.2			58.08		0.12
11	10	Axial	0	−1.68	0	35.88			36.98		−1.10
16	11	Center	0	0	0	59.11			58.08		1.03
7	12	Factorial	−1	1	1	36.95			36.67		0.28
9	13	Axial	−1.68	0	0	44.74			46.40		−1.66
10	14	Axial	1.68	0	0	59.01			56.41		2.60
3	15	Factorial	−1	1	−1	41.41			41.89		−0.48
4	16	Factorial	1	1	−1	50.65			51.09		−0.44
1	17	Factorial	−1	−1	−1	39.11			36.44		2.67
19	18	Center	0	0	0	58.21			58.08		0.13
2	19	Factorial	1	−1	−1	44.46			45.41		−0.95
6	20	Factorial	1	−1	1	40.93			41.12		−0.19
**Variable**					**Code**	**Coded and actual levels**					
						**−1.68**	**−1**	**0**		**1**	**1.68**
Incubation time (days)					X_1_	3.32	4	5		6	6.68
Inoculum size (mL/50 mL medium)					X_2_	2.32	3	4		5	5.68
Yeast extract (g/L)					X_3_	1.16	1.5	2		2.5	2.84

The CCD experimental data obtained from the 20 experiments were submitted to statistical analysis using design expert 7 software to determine the relationship between variables with the most significant positive effects and uricase production and the results are shown in Tables 5–7. The model *F*-value of 35.92 implies the model is significant. Our model has R^2^ = 0.9700 that mean 97% of the variability in uricase production by *Aspergillus welwitschiae* could be explained by the independent variables used and only 3% of the variability are not explained by the independent variables. This means that regression model provides an excellent explanation of the relationship between the independent variables and uricase production. In addition to the Adjusted R^2^ of 0.9430 was very high to prove the accuracy of the model to calculate the predicted uricase production. Also, the adjusted R^2^ shows a high correlation between the observed and the fitted values of uricase production. The value of C.V. (4.82%) indicates greatest accuracy of the experiment. In addition, PRESS value is 318.46 and the standard deviation and mean values are 2.25 and 46.64; respectively (Table 5).

**Table 5:** ANOVA for CCD results of uricase production by *Aspergillus welwitschiae*.

Source	Sum of Squares	*df*	Mean Square	*F-*value	*P-*value *P*rob >*F*
Model	1633.99	9	181.55	35.92	<0.0001
X_1_ - (incubation time)	121.05	1	121.05	23.95	0.0006
X_2_ - (inoculum size)	13.24	1	13.24	2.62	0.1366
X_3_ - (yeast extract)	77.16	1	77.16	15.27	0.0029
X_1_ X_2_	0.03	1	0.03	0.01	0.9413
X_1_ X_3_	19.59	1	19.59	3.88	0.0773
X_2_ X_3_	25.85	1	25.85	5.11	0.0473
X_1_^2^	80.21	1	80.21	15.87	0.0026
X_2_^2^	680.98	1	680.98	134.72	<0.0001
X_3_^2^	814.05	1	814.05	161.05	<0.0001
Residual	50.55	10	5.05		
Lack of Fit	38.22	5	7.64	3.10	0.1199
Pure Error	12.33	5	2.47		
Cor Total	1684.53	19			
**PRESS**	318.46	**Adeq Precision**			15.8864
**Std. Dev**.	2.25	**R** ^**2**^			0.9700
**C.V.%**	4.82	**Pred R** ^**2**^			0.8110
**Mean**	46.64	**Adj R** ^**2**^			0.9430

**Table 6:** Regression coefficients of CCD results of optimization of uricase production by *Aspergillus welwitschiae*.

Factor	Coefficient estimate	Standard error	95% CI Low	95% CI High
Intercept	58.08	0.92	56.04	60.12
X_1_ - (incubation time)	2.98	0.61	1.62	4.33
X_2_ - (inoculum size)	0.98	0.61	−0.37	2.34
X_3_ - (yeast extract)	−2.38	0.61	−3.73	−1.02
X_1_ X_2_	0.06	0.79	−1.71	1.83
X_1_ X_3_	−1.57	0.79	−3.34	0.21
X_2_ X_3_	−1.80	0.79	−3.57	−0.03
X_1_^2^	−2.36	0.59	−3.68	−1.04
X_2_^2^	−6.87	0.59	−8.19	−5.55
X_3_^2^	−7.52	0.59	−8.84	−6.20

**Table 7:** Fit summary for experimental results of CCD for optimization of uricase production by *Aspergillus welwitschiae*.

Model Summary Statistics					
Source	SD	R^2^	Adjusted R^2^	Predicted R^2^	PRESS
Linear	9.60	0.1255	−0.0384	−0.2648	2130.67
2FI	10.48	0.1525	−0.2386	−1.1963	3699.77
Quadratic	2.25	0.9700	0.9430	0.8110	318.46
**Lack of Fit Tests**					
**Source**	***SS***	***Df***	***MS***	***F-*** **value**	***P-*** **value**
Linear	1460.75	11	132.80	53.87	0.0002
2FI	1415.28	8	176.91	71.76	<0.0001
Quadratic	38.22	5	7.64	3.10	0.1199
Pure Error	12.33	5	2.47		
**Sequential Model Sum of Squares**					
Linear vs Mean	211.46	3	70.49	0.77	0.5298
2FI vs Linear	45.47	3	15.16	0.14	0.9355
Quadratic vs 2FI	1377.06	3	459.02	90.81	<0.0001
Residual	14.53	6	2.42		

“*Significant values, df: degree of freedom, PRESS: sum of squares of prediction error, 2FI: two factors interaction, SD: Standard deviation, SS: Sum of Squares, MS: Mean Square”.

The significance of each variable for uricase production was determined by *P*-value as listed in Table 5. It can be noted from the degree of significance that the linear coefficients of incubation time (X_1_) and yeast extract (X_3_) are significant. Also, the interaction between X_2_X_3_ and quadratic effects of the three variables are significant. On the other hand, among the model terms, the linear coefficients of inoculums size (X_2_), the interaction between incubation time (X_1_), inoculums size (X_2_) and the interaction between X_1_ X_3_ are not significant and not contribute to uricase production by *Aspergillus welwitschiae*. Furthermore, the quadratic effects of inoculums size and yeast extract were determined to have very significant effects on the production of uricase by *Aspergillus welwitschiae* with *P*-value < 0.0001.

Table 6 shows regression coefficients of the second order polynomial model for optimization of uricase production by *Aspergillus welwitschiae* and the equation of regression can be obtained in coded terms:

2 $$\begin{array}{c}{\bf{Uricase}}\,{\bf{activity}}\,=+58.08+2.98\ast {{\rm{X}}}_{1}+0.98\ast {{\rm{X}}}_{2}-2.38\ast {{\rm{X}}}_{3}+0.06\ast {{\rm{X}}}_{1}\ast {{\rm{X}}}_{2}-1.57\ast {{\rm{X}}}_{1}\ast {{\rm{X}}}_{3}\\ \,\,-1.80\ast {{\rm{X}}}_{2}\ast {{\rm{X}}}_{3}-2.36\ast {{\rm{X}}}_{1}^{2}-6.87\ast {{\rm{X}}}_{2}^{2}-7.52\ast {{\rm{X}}}_{3}^{2}\end{array}$$

where X_1_ is incubation time, X_2_ is inoculums size and X_3_ is yeast extract concentration.

The fit summary results are shown in Table 7, as the model summary statistics used to select the model which has higher adjusted and predicted R^2^ and lower standrd deviation. Table 7 shows that, the quadratic model is a high-significant and the proper model adequate for the CCD for *Aspergillus welwitschiae* uricase production with very low *P-*value (<0.0001) and the model also showed high lack of fit *F*-value = 3.10 (statistically insignificant lack of fit, *P-*value = 0.1199). The quadratic model shows the highest adjusted R^2^ (00.9430) and predicted R^2^ (0.8110). The quadratic model also shows the smallest standard deviation of 2.25.

### Contour and three dimensional (3D) plots

The 3D and Contour plots provide a way to visualize the relationship between the uricase activity and the interactions between the tested variables and to determine the optimal conditions for production of uricase. Three-dimensional plots were created for pairs of the three significant variables by plotting uricase activity against two variables and keeping the third variable at its zero level.

**Figure 5:**
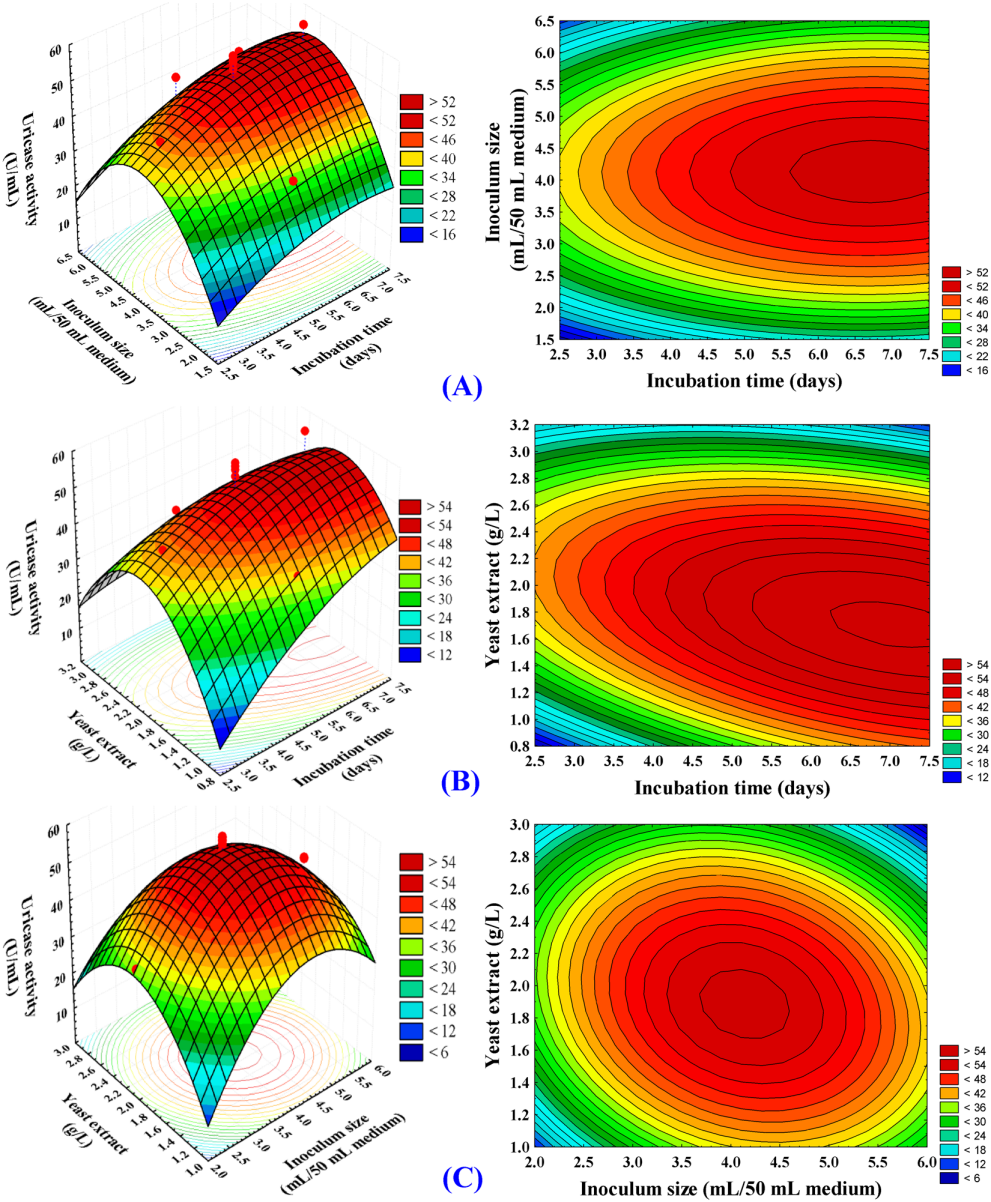
3D response surface and contour plots of the effects of incubation time (X_1_), inocluum size (X_2_) and yeast extract concentration (X_3_) and their mutual effect on the uricase activity.

Figure 5A shows the effects of incubation time and inoculums size on uricase production by *Aspergillus welwitschiae*, while the yeast extract concentration was kept at its zero level (2 g/L). Lower and higher levels of inoculums size cause relatively low production of uricase, the central point of inoculums size (4 mL/50 mL of the production medium) support the maximum uricase production. On the other hand, uricase production increases gradually with increasing incubation time. The highest activity was obtained after 5 days of incubation. By solving the Eq. (2) and analyzing Fig. 5A, the maximum predicted uricase activity of 59.03 U/mL was attained at the optimal predicted levels of incubation time (5.64 days incubation) and inoculum size (4.14 mL/50 mL of the production medium) at yeast extract concentration of 2 g/L.

Our results show that *Aspergillus welwitschiae* uricase activity was found to be 60.03 U/mL at inoculum size of 4 mL/50 mL of the production medium, but Hatijah and Ruhayu^34^ used 10 mL inoculum to produce uricase from *Aspergillus flavus* in 90 mL of medium. Nanda *et al*.^16^ used 100 μL inoculum of *Gliocladium virde* to produce uricase in 50 mL of production medium. The effect of inoculum size on the enzyme production is depending on the conditions of culture fermentation such as; nature of the used microbe, incubation time, and the characteristics of used substrate. Also, the fungal sporulation and its metabolic activities are affected by inoculums size^35^. If the inoculum size is low, it may take longer time to multiply the fungal cells and utilize the substrate to produce the desired enzyme. On the other hand, a high inoculum would create a rapid reproduction of fungal biomass. Consequently, the balance between substrate utilization and proliferating biomass would give maximum enzyme activity as demonstrated by Sherief *et al*.^36^. Increase in inoculum size resulted in decreasing enzyme yield due to limitation of nutrients as reported by El-Naggar *et al*.^37^.

The second significant factor is the incubation time which significantly affects the enzyme production. In our study the optimum incubation time for maximum uricase production by *Aspergillus welwitschiae* was 5 days. These results are consistent with the results of Kon *et al*.^38^ who reported that the maximum uricase produced by *Hyphomyces* was obtained after 5 days of incubation. On the other hand, Abdel-Fattah and Abo-Hamed^39^ reported that uricase was produced from *Aspergillus flavus*, *Aspergillus terreus* after 4 days of incubation and from *Trichoderma* sp. after 6 days. In contrast, Nanda *et al*.^16^ proved that *Gliocladium viride* MTCC 3835 was identified to produce highly active uricase (82.1 U/mL) after 7 days of incubation. Our finding is inconsistent with the results of Atalla *et al*.^40^ who reported that the incubation for 8 days was required for maximum production of uricase by *Gliomastix gueg*. Moreover, Nour El-Dein and El- Fallal^41^ found that uricase was produced by *Aspergillus carbonarius*, *Botrytis fabae*, *Aspergillus sydowi* after 10 days of incubation. The maximum amount of intracellular uricase produced by *Mucor hiemalis* in a simple medium was achieved in 24 hours^19^. Jagathy *et al*.^42^ reported that the maximum production of uricase (141 U/mL) by *Aspergillus niger* was obtained at 96 hours of incubation.

Figure 5B presented the effects of incubation time and yeast extract on uricase production while the inoculums size was retained at its zero level (4 mL/50 mL of the production medium). The maximum production of uricase clearly obtained near the central level of yeast extract concentration (2 g/L) while the higher and lower levels of the yeast extract lead to a lower rate of uricase production. On the other hand, uricase production increases gradually with increasing incubation time and the maximum production of uricase was obtained after 5 days of incubation. By solving the Eq. (2) and analyzing Fig. 5B, the maximum predicted uricase activity (59.41 U/mL) was attained at the optimal predicted levels of incubation time (5.68 days of incubation) and yeast extract concentration (1.89 g/L) at inoculum size of 4 mL/50 mL of the production medium.

The fungal enzymes production is very sensitive to nitrogen source and its level in the medium. Generally, the use of organic nitrogen increases uricase production significantly more than inorganic nitrogen. Organic nitrogen may contain most of the amino acids necessary for microbial growth, which can be directly metabolized by cells to promote the production of uricase. Yeast extracts contain different biologically active compounds, including nucleotides and polysaccharides of the cell wall (especially α-mannan and β-glucan) that are useful for enzyme production. It may be acting as medium components that induce uricase production. Yeast extract is considering one of the effective variables on uricase production. Our results have shown that the optimal concentration of yeast extract for uricase production was 2 g/L. Our findings were agreed with the results obtained by Anderson and Vijayakumar^43^ who used 2 g/L yeast extract for uricase production by *Pseudomonas aeruginosa*. Abbas^44^ also reported that the highest value of specific activity of uricase (14.83 U/mg) produced by *Asperigillus niger* was obtained using 2% of the yeast extract. However, there is disagreement with Nanda *et al*.^16^ who proved that the best concentration of the yeast extract was 10.57 g/L for maximum uricase production (82.1 U/mL) by *Gliocladium viride* MTCC 3835. The optimal yeast extract concentration for maximum uricase production (0.23 U/mL) by *Pseudomonas* sp. was 0.5%^45^. Khucharoenphaisan and Sinma^46^ reported that 1% yeast extract as a nitrogen source increased uricase production by *Saccharopolyspora* sp. PNR11 up to 216 mU/mL.

Figure 5C shows the interaction between inoculums size and yeast extract concentration while incubation time was kept at its zero level. By increasing the inoculums size to 4 mL/50 mL of the production medium and yeast extract concentration to 2 g/L, the maximum production of uricase was obtained. By solving the Eq. (2) and analyzing Fig. 5C, the maximum predicted uricase activity of 58.31 U/mL was attained at the optimal predicted levels of inoculums size of 4 mL/50 mL of the production medium and yeast extract concentration of 1.91 g/L at incubation time of 5 days.

Finally, the maximum production of uricase by *Aspergillus welwitschiae* was found to be 60.03 U/mL where uric acid concentration used was 4 g/L, yeast extract 2 g/L and inoculums size 4 mL at the 5^th^ day of incubation at 35 °C in 50 mL production medium. Our results are higher than the results of Nour El-Dein and El-Fallal^41^ who found that *Aspergillus carbonarius* produced uricase with activity of 0.16 U/mL/min and *Botrytis fabae*, *Aspergillus sydowi* produced 0.13, and 0.093 U/mL; respectively. While *Aspergillus terreus*, *Aspergillus niger* and *Aspergillus alutaceus* produced below 0.06 U/mL in medium consisted of (% w/v): sucrose, 3; KH_2_PO_4_ 0.1; Mg SO_4_. 7H_2_O 0.05; NaNO_3_ 0.2; uric acid 2.0 and pH 7.4. 50 mL of the liquid medium was inoculated with different fungal strains and incubated at 30 °C or 25 °C for ten days. Yazdi *et al*.^19^ found that *Mucor hiemalis* produced 1.25 U/mL, where uric acid concentration was 7.0 g; the optimum temperature and pH were 30 °C and 6; respectively in 50 mL medium/250 mL conical flask. On the other hand, Nanda *et al*.^16^ proved that *Gliocladium viride* MTCC 3835 was identified to produce highly active uricase (82.1 U/mL**)** using the optimal concentrations of variables which were yeast extract 10.57 g/L, peptone 12.71 g/L, uric acid 2 g/L, CuSO_4_ 0.0762 g/L and pH 7.5. The inoculated medium was incubated for 7 days at 30 °C in 50 mL production medium.

### Model verification

For determination the model accuracy and for verification of the results, an experiment was performed under the optimal conditions obtained from CCD. The experimental uricase activity was 60.03 U/mL which was close to the predicted value of 58.08 U/mL. This revealed a high degree of precision (96.75%).

## Materials and Methods

### Microorganism and culture maintenance

*Aspergillus* sp. strain 1–4 that used in this study was newly isolated by the fourth author from soil sample collected from Egypt and cultured on Petri plates containing potato dextrose agar (PDA) medium that composed of potato infusion (infusion from 200 g potatoes), 20 g dextrose, and 15 g agar. After the incubation at 30 °C for 5 days, the plates were maintained at 4 °C for further use.

### Extracellular uricase production potential of *Aspergillus* sp. strain 1–4

The fungal strain was examined for its potential to produce uricase by agar plate assay method on the solidified medium which contains uric acid as an inducing agent. Uric acid screening medium was prepared using the following components (g/L): uric acid 3, sucrose 20, magnesium sulphate heptahydrate 0.5, sodium chloride 0.5, di-potassium hydrogen phosphate 1, ferrous sulphate 0.01 and agar 15^39^. The pH was adjusted to 6.8 and the inoculated plates were incubated for 5–7 days at 30 °C. Clear zone formed around the fungal colony indicate a positive result for production of uricase. The ability of *Aspergillus* sp. strain 1–4 to produce uricase was then confirmed under submerged fermentation.

### Inoculum preparation and submerged-fermentation

Fifty mL of liquid uric acid production medium of the following composition (g/L): sucrose 20, uric acid 3, NaCl 0.5, K_2_HPO_4_ 1, MgSO_4_.7 H_2_O 0.5, Fe_2_SO_4_ 0.01, distilled water added up to 1 L, pH adjusted to 6.8, in 250 mL Erlenmeyer flask was inoculated by five discs from five days old stock culture of *Aspergillus* sp. strain 1–4. The inoculated flask was incubated at 30 °C for 48–72 h, and was used as inoculum for subsequent experiments. The production medium was inoculated with the prepared inoculum and incubated at specified temperature (25–35 °C). After the specified incubation time for each set of experimental trials, the mycelia of the strain 1–4 were collected by centrifugation at 5000 × *g* for 15 min and the cell free supernatant was used as a crude enzyme preparation for further determinations of the enzymatic activity.

### Uricase assay

According to Adamek *et al*.^9^ procedures, uricase activity was assayed. Assay mixture containing two mL of buffer solution containing uric acid (0.06 mM uric acid in 0.2 M sodium borate buffer, pH 8.5), 0.8 mL of distilled water and 0.1 mL of crude enzyme preparation was incubated at 37 °C in water bath for 30 min. After 30 min of incubation, 0.2 mL of 0.1 M potassium cyanide solution was added to the mixture to stop the enzyme reaction. The blank was prepared by adding potassium cyanide solution before adding the uricase. The absorbance was measured spectrophotometrically against the blank at 293 nm. The difference between the absorbance of the sample and the blank is directly proportional to the decrease in uric acid concentration throughout uricase reaction. One unit of uricase is defined as the amount of the uricase that produces 1 µmol of hydrogen peroxide per minute under the assay conditions.

### Scanning electron microscopy

Scanning electron microscopy is a useful tool for studying fungi. It allows the study of several aspects of morphology^47^. The gold-coated dehydrated fungal growth specimen was examined at different magnifications using “Analytical Scanning Electron Microscope (JSM-6510 LV) at Electron Microscope unit, Mansoura University, Mansoura, Egypt”.

### Molecular identification of *Aspergillus welwitschiae* and phylogenetic analysis

The preparation of genomic DNA of *Aspergillus* sp. strain 1–4 was conducted in accordance with the methods described by Sambrook *et al*.^48^. DNA extraction, polymerase chain reaction (PCR) and 18S rRNA sequencing were performed by Macrogen Korea Company Gasan-dong, Geumchen-gu, Seoul, Korea (http://www.macrogen.com). The amplification of 18S rRNA gene from *Aspergillus* sp. strain 1–4 was carried out via PCR. Primers used were reverse primer ITS4 “5′-TCCTCCGCTTATTGATATGC-3′” and forward primer ITS1 “3′-TCCGTAGGTGAACCTGCGG-5′”. The amplification was carried out in 100 μl contained: “1 μl DNA, 10 μl of 250 mM dNTP’s; 10 μl PCR buffer, 3.5 μl 25 mM MgCl_2_ and 0.5 μl Taq polymerase, 4 μl of 10 pmol (each) forward and reverse primer and water was added up to 100 μl”. Components of the PCR reaction were mixed thoroughly. DNA amplification was carried out in the thermal cycles using the following PCR programme: 10 min denaturation at 95 °C, then 30 sec of 35 amplification cycles at 95 °C, annealing of 1 min at 55 °C, extension of 1 min at 72 °C, and 15 min final extension at 72 °C, number of cycles equal 35. The PCR reaction mixture was purified using purification Kit of Thermo (GeneJET™ PCR, K0701) K0701.

### Phylogenetic analysis

The obtained sequences of 18S rRNA gene for *Aspergillus* sp. strain 1–4 was analyzed using basic local alignment search tool (BLAST)^26^ (https://blast.ncbi.nlm.nih.gov/Blast.cgi?PROGRAM=blastn&PAGE_TYPE=BlastSearch&LINK_LOC=blasthome) at NCBI database and the obtained sequence was compared with the 18S rRNA related sequences of representative members of fungi retrieved from the Gen Bank, DDBJ, PDB and EMBL databases. The phylogenetic tree was constructed via the neighbor-joining algorithm^27^ using the software package MEGA4 version 2.1^49^.

### Screening of various process factors influences uricase production by Plackett–Burman design

Plackett-Burman technique was used to screen the fermentation medium components and the environmental conditions to identify the foremost important variables that had a significant impact on the production of uricase. The statistical experimental design of Plackett and Burman^50^ is very useful in identifying the most significant variables with regard to their main effects^51^. Plackett–Burman statistical experimental design does not describe the interaction between variables and is used only to screen and evaluate the important variables that affect the response^52^. A number of tools may be used to help assessing the significance of each process factor. This includes normal plots, *P*-values, and Pareto charts.

Based on the Plackett-Burman factorial design, the nutritional and environmental requirements of *Aspergillus* sp. strain 1–4 for uricase production were examined in two levels which were low level (−1) and high level (+1). In our study, fifteen independent variables were examined for selection of significant variables for uricase production; these variables included different energy sources, nitrogen sources, carbon sources, metals and physical variables. In addition, four unassigned dummy variables (D_1_–D4) were used for estimation of the experimental errors in data analysis.

Plackett–Burman experimental design is based on the equation of the first order polynomial model:

3 $${\rm{Y}}={\beta }_{0}+\sum {{\beta }}_{{\rm{i}}}{{\rm{X}}}_{{\rm{i}}}$$

where, Y is the activity of uricase; β_0_ is the intercept of the model; *β*_*i*_ is the linear coefficient and X_i_ is the independent factors levels. All experiments were performed in duplicate and the responses were considered to be the average uricase activity.

### Optimization of uricase production by using central composite design (CCD)

Based on the results of Plackett-Burman experiment, the three variables with most significant positive effect on uricase production by *Aspergillus* sp. strain 1–4 and had the highest percentage of contribution were further optimized using CCD. These variables were coded as X_1_, X_2_ and X_3_ and examined in 5 levels which were −1.68, −1, 0, +1, +1.68. According to the CCD design, combinations of the three independent variables were conducted in twenty experiments and the results were fitted to the following equation of second order polynomial model:

4 $${\rm{Y}}={{\beta }}_{0}+{\rm{\sum }}_{{\rm{i}}}{{\beta }}_{{\rm{i}}}{{\rm{X}}}_{{\rm{i}}}+\sum _{\mathrm{ii}}{{\beta }}_{\mathrm{ii}}{{\rm{X}}}_{{\rm{i}}}^{2}+{\rm{\sum }}_{\mathrm{ij}}{{\beta }}_{\mathrm{ij}}{{\rm{X}}}_{{\rm{i}}}{{\rm{X}}}_{{\rm{j}}}$$

Y is the predicted uricase activity, β_i_ is the linear coefficient, β_0_ is the regression coefficients, X_i_ is the coded levels of the independent variables, β_ij_ is the interaction coefficients and β_ii_ is the quadratic coefficients.

### Statistical analysis

The obtained results were subjected to multiple linear regression analysis using “Design Expert software version 7 (Stat-Ease Inc., USA) for Windows. The statistical software package, STATISTICA software (Version 8.0, StatSoft Inc., Tulsa, USA) was used to plot the three-dimensional surface plots”.

## Conclusion

Different fifteen nutritional and environmental variables were screened for their significances on uricase production by *Aspergillus welwitschiae* using Plackett-Burman statistical design. Incubation time, inoculum size and yeast extract concentration identified by Plackett-Burman design as the most significant variables affecting positively uricase production by *Aspergillus welwitschiae* and were further optimized using central composite design. By using CCD, the optimal levels for these variables were: 5 days of incubation time, 2 g/L yeast extract and inoculums size 4 mL for maximum production of uricase by *Aspergillus welwitschiae* (60.03 U/mL), when 4 g/L uric acid, 50 mL production medium/250 mL conical flask were used and incubated at 35 °C. Uricase production in the optimized medium was increased up to 3.02- times compared to the initial medium of production. Our future studies will be will focus on semi-industrial production, purification and characterization of uricase along with its pharmacological characteristics, which will be very helpful when designing a novel strategy for the treatment of certain diseases such as chronic gout, tumor lysis hyperuricemia and organ transplants hyperuricemia.

## Supplementary information

Supplementary Information
