# Supplementary material for: Mathematical modeling for bioprocess optimization of a protein drug, uricase, production by Aspergillus welwitschiae strain 1–4
Source: Sci Rep. 2019 Sep 10;9:12971. doi: 10.1038/s41598-019-49201-1 (PMC6736946; doi:10.1038/s41598-019-49201-1)

Original Article

Mathematical modeling for bioprocess optimization of a protein drug, uricase, production by *Aspergillus welwitschiae* strain 1-4

[1]Noura El-Ahmady El-Naggar, [2]S.A. Haroun, [2] Eman M. El-Weshy, [2] E.A. Metwally, and [2]A.A. Sherief

[1] Department of Bioprocess Development, Genetic Engineering and Biotechnology Research Institute, City of Scientific Research and Technological Applications, Alexandria 21934, Egypt

[2] Department of Botany, Faculty of Science, Mansoura University, Egypt

Correspondence should be addressed to:

Prof. Noura El-Ahmady Ali El-Naggar

Address:

Bioprocess Development Department,
Genetic Engineering and Biotechnology Research Institute,
City of Scientific Research and Technological Applications,
New Borg El- Arab City, 21934, Alexandria, Egypt

Tel: (002)01003738444

Fax: (002)03 4593423

E-mail: nouraelahmady@yahoo.com

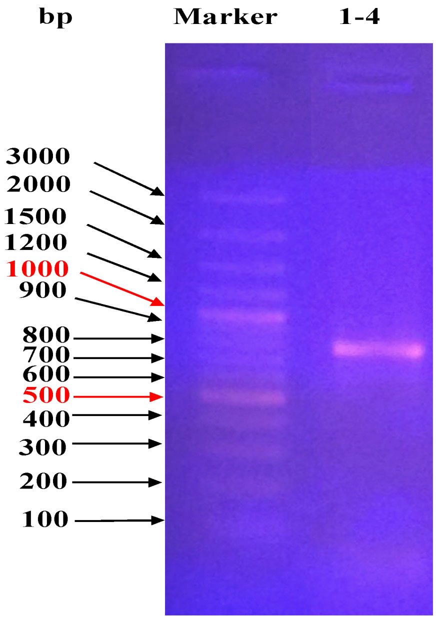

Supplementary Figure S1. Agarose gel electrophoresis shows the PCR product of the amplified *Aspergillus welwitschiae* 18S rRNA fragment.

Complete gel for Figure S1

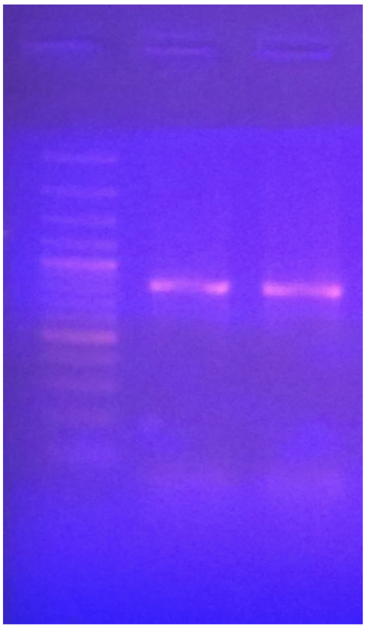

Supplement: Supplementary file 1 — Supplementary Information [file 41598_2019_49201_MOESM1_ESM.pdf]
